# A Precise Microfluidic Assay in Single‐Cell Profile for Screening of Transient Receptor Potential Channel Modulators

**DOI:** 10.1002/advs.202000111

**Published:** 2020-04-19

**Authors:** Xiaoni Ai, Yang Wu, Wenbo Lu, Xinran Zhang, Lin Zhao, Pengfei Tu, KeWei Wang, Yong Jiang

**Affiliations:** ^1^ State Key Laboratory of Natural and Biomimetic Drugs School of Pharmaceutical Sciences Peking University Beijing 100191 China; ^2^ Department of Pharmacology Qingdao University School of Pharmacy 38 Dengzhou Road Qingdao 266021 China

**Keywords:** drug screening, microfluidics, pain relief, single‐cell analysis, transient receptor potential channel modulators

## Abstract

Transient receptor potential (TRP) channels are emerging drug targets, and TRP channel modulators possess therapeutic potential for many indications. However, there is a lack of intellectual and robust screening assays against TRP channels utilizing the least amount of compounds. Here, a precise microfluidic assay in single‐cell profile is developed for the screening of TRP channel modulators. The geometrically optimized microchip is designed for both trapping single cells and utilizing passive pumping for sequential media replacement with low shear stress. The microfluidic chip exhibits superior performance in screening, repeatable compound administration, and improved reproducibility. Using this screening platform, the false‐positive and negative rate of the commonly used Ca^2+^ imaging is reduced from 76.2% to 4.8% and four coumarin derivatives isolated from *Murraya* species that inhibit TRP channels are identified. One coumarin derivative B‐304 reverses TRPA1‐mediated inflammatory pain in vivo. Taken together, the data demonstrate that the established microfluidic assay in single‐cell profile could be used for the screening of TRP channel modulators that may have therapeutic potential for the channelopathies.

## Graphical abstract

A precise microfluidic assay in single‐cell profile for screening of transient receptor potential (TRP) channel modulators is developed. The single‐cell‐based screening strategy dramatically reduces false‐positive/negative results from 76.2% to 4.8% due to full consideration of single‐cell heterogeneity. Four novel coumarin derivatives are cherry‐picked to potently inhibit TRP channels. One of the positive hits, B‐304, reverses TRPA1‐mediated inflammatory pain in vivo.

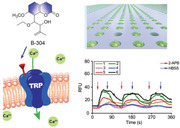

## 1 Introduction

The mammalian transient receptor potential (TRP) channels mainly consist of six main subfamilies,^[^ ^1^, ^2^ ^]^ and most of them are non‐selectively permeable to monovalent and divalent cations, including Na^+^, Ca^2+^, and Mg^2+^.^[^ ^3^, ^4^ ^]^ Lines of evidences reveal that malfunctioning of TRP channels is involved in various human diseases,^[^ ^5^, ^6^ ^]^ rendering TRP channels as emerging therapeutic targets for drug discovery.^[^ ^6^, ^7^, ^8^ ^]^ Current available chemical modulators of TRP channels are either poorly selective or less potent. For instance, there is still no specific modulator known for TRPV2 channel, and the currently available activators of TRPV3 are characterized by low potency ranging from 20 µm to 100 mm.^[^ ^9^, ^10^ ^]^ Among TRP channels, TRPA1, TRPV1–4, and TRPM8 are located in nociceptors where they play crucial roles in detecting and transducing signals of thermal, chemical, and mechanical stimuli in pathological pain conditions.^[^ ^11^, ^12^ ^]^ Therefore, discovery and development of effective and selective TRP channel modulators may hold great value for pain treatment and the channel pharmacology.

There are two commonly used methods for screening of TRP channel modulators, indirect optical techniques based on Ca^2+^ imaging and direct functional patch‐clamp electrophysiology.^[^ ^13^, ^14^, ^15^ ^]^ The commercially available automated Ca^2+^ imaging apparatus of FlexStation 3 measures the intracellular fluorescent calcium signaling from population cells, resulting in an inaccurate prediction for false‐positive/negative hits.^[^ ^16^ ^]^ Patch‐clamp‐based single‐cell recording still remains as the gold standard for ion channel target‐based drug discovery.^[^ ^17^ ^]^ However, patch‐clamp recording assay is not suitable for large‐scale drug screening because of its relatively large amount of sample consumption and labor‐intensive and sophisticated micromanipulations. Single‐cell analysis has been extensively studied in recent years due to technical advances in single‐cell isolation^[^ ^18^, ^19^, ^20^ ^]^ and precise control of single‐cell microenvironment.^[^ ^21^, ^22^, ^23^ ^]^ Especially, microfluidics is a powerful methodology for single‐cell analysis with high‐throughput and spatiotemporal resolution.^[^ ^24^, ^25^ ^]^ Microfluidic techniques developed previously have shown efficient single‐cell capture,^[^ ^26^, ^27^ ^]^ in particular, the gravity‐based capture mechanism using microwells for trapping cells and delivering stimuli under low shear‐stress conditions.^[^ ^28^ ^]^ Shear stress has potential impact on cell viability and TRP channel functionality.^[^ ^29^ ^]^ Some microfluidic designs are featured of a chemical delivery module for accurate detection of single‐cell response dynamics.^[^ ^30^, ^31^ ^]^ Open microfluidics often requires more amounts of stimuli and is subjected to high risk of evaporation in comparison with closed microfluidics.^[^ ^32^ ^]^ On‐chip pneumatic valve precisely controls dynamic stimuli delivery; however, it involves complicated device manufacture and operation.^[^ ^23^ ^]^ Operationally simple approaches of gas/liquid exchange and two Y‐shaped channels with pinch valves may have issues with trace leakage.^[^ ^22^ ^]^ To our knowledge, there is no microfluidic approach especially established for target‐based compound screening in single‐cell format. The methodology of screening TRP channel modulators requires precise, low‐cost, and operationally simple delivery of stimuli with low shear stress, as well as simultaneous monitoring of single cells in a high‐throughput manner.

Here, we report the development of a single‐cell‐based strategy for screening of TRP channel modulators, especially from natural products that have contributed much success and progress in TRP channel research and drug discovery.^[^ ^33^ ^]^ We designed and fabricated a geometrically optimized microchip that traps single cells with passive pumping for precise, low‐cost, and operationally simple delivery of stimuli under low shear‐stress conditions. Furthermore, known TRP channel agonists and antagonists were used for the methodological validation of the unique microfluidic chips. Moreover, we screened ≈200 natural small molecules against cells individually expressing five different TRP channel subtypes. The on‐chip screening results were compared with FlexStation 3 and patch‐clamp assays. Finally, we assessed a TRPA1 antagonist B‐304 from the plant *Murraya* species and tested its effect on formalin‐ and allyl isothiocyanate (AITC)‐evoked pain behaviors in mice.

## 2 Results

### 2.1 Microchip Design and Single‐Cell Trapping

**Figure 1:**
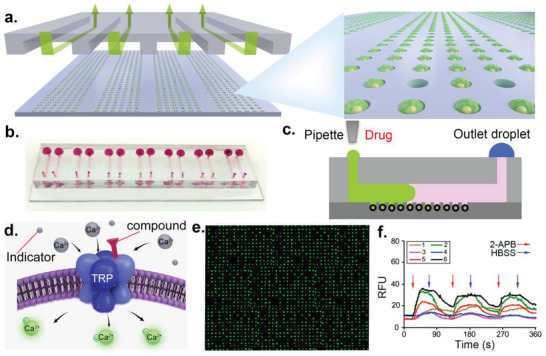
Schematics of the microfluidic assay in single‐cell profile for real‐time monitoring of calcium fluorescence kinetics upon active compounds targeting TRP channels. a) Schematic diagram of representative four microchips integration for single‐cell trapping. Green arrows indicate fluid direction. b) Photograph of 14 paralleled microchips’ integration on a glass slide. c) Schematic diagram of switching media by passive pumping on one microchip. d) Detection principle of TRP channel activity upon compound administration. e) Fluorescent image from hundreds of individual cells expressing TRP channel from one microchip. f) Kinetics of calcium fluorescence from the representative cells with repeated compound administration by passive pumping.

The single‐cell microfluidic device comprises densely arrayed traps within a closed flow‐through channel (**Figure** **1**a). A photograph of 14 paralleled microchips integration on a glass slide is shown in Figure 1b. The integration of multiple microchips improves the throughput and versatility of the platform. Passive pumping is used for media exchange (Figure 1c). Upon active compounds targeting TRP channels, the intracellular fluorescence intensity increases through Ca^2+^‐dependent influx with the indicator (Figure 1d).^[^ ^34^ ^]^ In our device, hundreds of the fluorescent calcium changes in single cells were tracked in a real‐time (Figure 1e,f).

The fundamental principle used for single‐cell trapping is a size‐based isolation. Parameters were optimized in order to obtain the highest single‐cell capture efficiency through microwell spacing, microwell diameter, cell density seeding, and loading times (Figure S1, Supporting Information). Human embryonic kidney (HEK)‐293T cells and Chinese hamster ovary (CHO) cells are of low background and are commonly used in the screen of ion channels, including TRP channels. These cell lines are also quite stable over long‐term cultures and passages and are easily transfected with high efficiencies. We measured the diameter of the HEK‐293T cells with 15.8 ± 2.2 µm, similar to that of CHO cells at 15.7 ± 0.7 µm. Due to size‐based trapping, we chose the HEK‐293T cells for parameter optimization because of higher transfection efficiency with higher membrane protein yields than CHO cells. To acquire more than 300 fluorescence dynamics of single cells in one screen test using microscopy with a 10× objective, we chose the microwell design with 25 µm diameter, 40 µm depth, and 60 µm spacing, as well as the cell seeding density at 1 × 10^7^ cells mL^−1^ for a 1st round cell loading, and an additional two rounds of cell loading with a density of 5 × 10^6^ cells mL^−1^. With the optimized parameters, the single‐cell trapping efficiency reached 86.4% ± 3.6%, and the percentages of microwells with empty and multiple cells were 3.7% ± 0.9% and 9.9% ± 1.2%, respectively.

### 2.2 Media Replacement by Passive Pumping

Due to hard‐to‐get and trace properties of natural products, we designed a closed microchannel for fast media replacement with minimum compound consumption and evaporation. Media replacement is achieved by surface tension between the ports at both ends of the closed channel (Figure 1c). According to the Young–Laplace equation, smaller drops have higher internal surface energy than larger drops to drive the liquid through the microchannel (Figure S2a, Supporting Information). We, therefore, chose the smaller drop in 4 µL and larger drop in 30 µL for media replacement, which was sufficient to fill the closed microchannel and completely replace the previous liquid within 8 s at a flow rate of 0.49 µL s^−1^ (Figure S2b, Supporting Information). The operation of media replacement had no physiological damage to the cells (Figure S2c, Supporting Information). The computational analysis also illustrated that the shear stress in the deep microwell was much lower than that in the microchannel (Figure S2d, Supporting Information). Therefore, the microwell retained cells without being flushed away during media replacement. The results also indicated that cells trapped in the microwells were exposed to low shear stress minimally perturbing cell viability and TRP channel functionality. The passive pumping used in this work represents a tube‐less form of media replacement with no requirement of specific microfluidic skills. We also tried other methods for media replacement, such as gas/liquid exchange and two Y‐shaped channels with pinch valves. However, only the passive pumping was efficient without leakage‐induced premature activation/inhibition of TRP channels. Therefore, the microfluidic platform integrated functionality of passive pumping, closed microchannel and microwell traps that facilitated precise, least amount of compounds, and operationally simple delivery of stimuli with low shear stress.

### 2.3 Methodological Validation of the Microchip Using Tool Compounds Screening Against TRP Channels

Herein, we evaluated the feasibility of the microfluidic chip for precise monitoring of fluorescent calcium dynamics at the single‐cell level. Non‐selective agonist and antagonist of TRP channels, 2‐aminoethoxydiphenyl borate (2‐APB) and ruthenium red (RR), were used for demonstrating the capability of the microchip for monitoring their effects on different TRP channel subtypes.^[^ ^35^, ^36^ ^]^ Cells functionally expressing TRP channels could be activated by agonists, as compared with non‐transfected cells that exhibited no currents (Figure S3, Supporting Information). More than 300 fluorescence changes of individual TRP‐transfected cells were tracked before and during the application of tool compounds.

**Figure 2:**
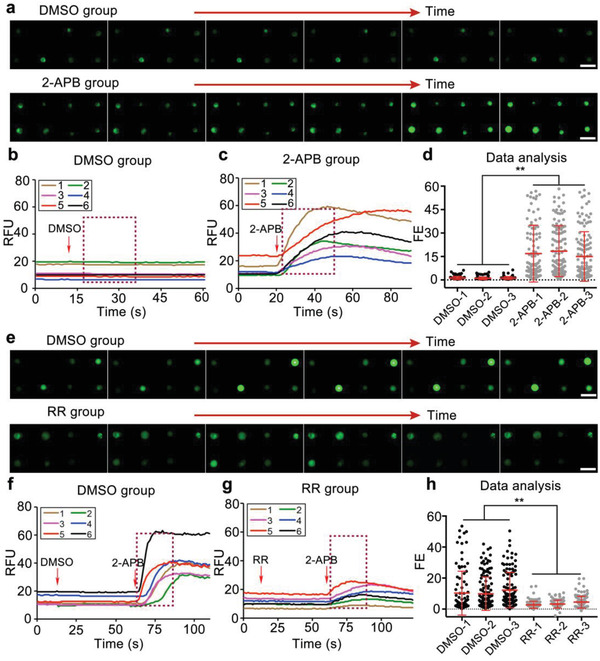
Methodological validation of the microchip for monitoring intracellular calcium response of individual cells to a–d) TRPV1 agonist 2‐APB and e–h) antagonist RR. a,e) Magnified fluorescent images from hundreds of TRPV1‐transfected cells in the DMSO groups and the compound groups over time. Representative kinetics of calcium fluorescence from hundreds of individual cells in the DMSO groups (b,f) and the compound groups (c,g). d,h) Fluorescence enhancement distribution (FE) from hundreds of individual cells exhibited distinct cell heterogeneity. The fluorescent images and kinetics of the representative cells were randomly chosen from at least 300 individual cells. Scale bar: 50 µm. Values are presented as means ± S.D. from independent experiments performed in triplicate. **p < 0.01, relative to control group.

First, we examined the feasibility of the microfluidic chip for real‐time monitoring of the intracellular calcium change in response to the agonist 2‐APB for TRPV1 and TRPV3. The magnified fluorescence intensity from hundreds of cells was elevated upon channel activation (**Figure** **2**a). The fluorescent calcium kinetics of individual cells exhibited distinct cell heterogeneity, as indicated by the large deviation in six fluorescence profiles of the representative cells (Figure 2b,c). Owing to the visualization of the transient calcium signal, we eliminated ≈15–35% of the cells without fluorescence response possibly due to low transfection efficiency. We further calculated the fluorescence enhancement from hundreds of individual cells in which non‐selective agonist 2‐APB activated TRPV1 and TRPV3 channels with strong fluorescence enhancement, exhibiting heterogeneity that can otherwise be overlooked in population cells (Figure 2d and Figure S4a, Supporting Information).

Next, we investigated the feasibility of the microfluidic chip for real‐time monitoring of the intracellular calcium levels in response to the antagonist RR for TRPV1 and TRPV3 (Figure 2e–h and Figure S4b, Supporting Information). Compared with the DMSO exposure, the fluorescence increase was inhibited dramatically by RR. The representative dynamic fluorescence profiles and the scatter plot of the fluorescence enhancements from hundreds of single cells showed variations, and the inhibition rates of TRPV1 and TRPV3 channels by 20 µm RR were calculated to be 67.7% ± 8.3% and 54.9% ± 8.9%, respectively (*n* = 3), consistent with a previous report.^[^ ^35^ ^]^ The results indicated that the single‐cell arrayed microchip was capable for real‐time monitoring the change of intracellular calcium in response to TRP channel modulators.

### 2.4 Superior Performance of the Microchip for Monitoring TRP Channel Activity

**Figure 3:**
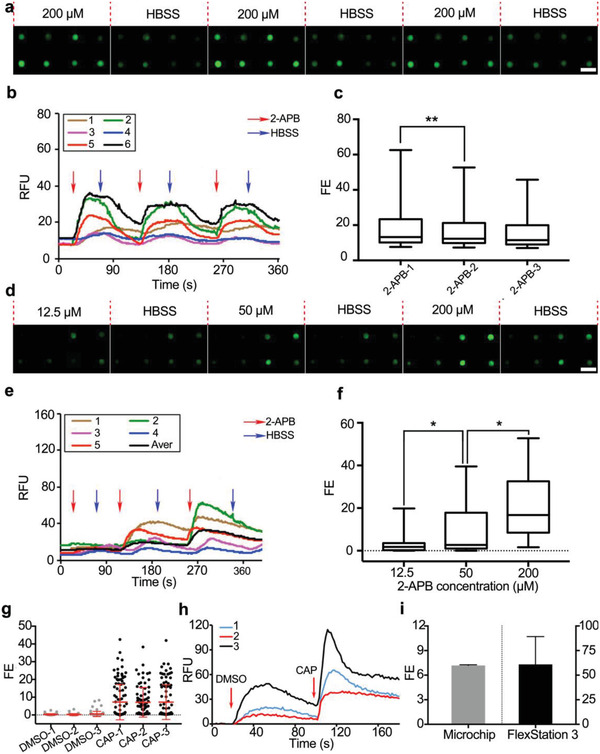
Superior performance of the microchip for monitoring TRP channel activity. a–f) Repeated compound administration by passive pumping: a,d) Magnified fluorescent images from hundreds of TRPV3‐transfected cells with a) the repetitive applications of 200 µm 2‐APB, as well as d) the increasing doses of 2‐APB with 12.5, 50, and 200 µm. b,e) Representative kinetics of calcium fluorescence and c,f) boxplots of the fluorescence enhancement distribution from hundreds of individual cells. g–i) Comparison of reproducibility of results from microfluidic assay and FlexStation 3 assay. The fluorescent images and kinetics of representative cells were randomly chosen from at least 300 individual cells. Scale bar: 50 µm. Values are presented as means ± S.D. from independent experiments performed in triplicate. *p <0.05, **p < 0.01, relative to control group.

The conventional Ca^2+^ imaging method such as FlexStation 3 assay is unable to accommodate repeated compound applications due to the lack of elution process. To test the capability of the microfluidic chip for repetitive compound administrations through passive pumping, we conducted repetitive applications of 2‐APB and observed a dose‐dependent activation of TRPV3 channel with an EC_50_ value at 35.20 µm (**Figure** **3**a–f), consistent with a previous report of 28.3 µm at +80 mV and 41.6 µm at −80 mV.^[^ ^37^ ^]^ This repeated compound application feature by passive pumping provides a practical potential in studying sensitization or desensitization of TRP receptors,^[^ ^38^, ^39^ ^]^ as well as their pharmacology.

We further compared the fluorescence signals obtained from the on‐chip individual cells and the population cells using FlexStation 3 assay (Figure 3g,h). The analysis of the fluorescence enhancements revealed a significantly smaller standard deviation from the microfluidic method in comparison to FlexStation 3 assay (Figure 3i).

Therefore, the microfluidic chip exhibits unique advantages in compound screening, repeated compound administration, and improved data reproducibility due to passive pumping and hundreds of single‐cell profile in one screen test.

### 2.5 Screening of Small Molecule Modulators Targeting TRP Channel

A library of ≈200 small molecules was screened against cells expressing five TRP channel subtypes (TRPA1 and TRPV1–4). The total size of screening samples was about 1000. The library mainly comprised compounds separated from *Dictamnus dasycarpus* and *Murraya* plants, which have been widely used as medicinal herbs for remedies in skin diseases and pain relief.

The conventional Ca^2+^ imaging of FlexStation 3 assay was first used for a primary screening. A batch of active compounds was obtained and their activities are listed in Table S1 and Figure S5, Supporting Information. We next utilized the single‐cell arrayed microchip to perform a secondary screening, followed by the confirmation using the gold standard of patch‐clamp recordings. Because of high reproducibility of measurements obtained by the microfluidic method as mentioned above, it was hopeful that the single‐cell arrayed microchip could greatly increase the accuracy of screening.

**Figure 4:**
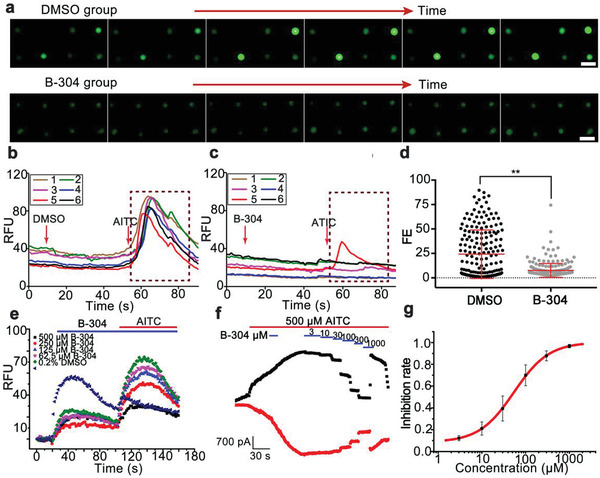
The novel coumarin B‐304 compound inhibited the TRPA1 channel. a) Magnified fluorescent images from hundreds of TRPA1‐transfected cells in the DMSO group and the B‐304 group over time. b,c) Representative kinetics of calcium fluorescence from hundreds of individual cells in b) the DMSO group and c) the B‐304 group. d) On‐chip fluorescence enhancement distribution of hundreds of the individual cells from the DMSO group and the B‐304 group. e) Fluorescent traces of the dose‐dependent B‐304 inhibition through TRPA1 channel using the FlexStation 3 assay. f,g) The validation of the B‐304 inhibition on TRPA1 channel by patch‐clamp. The whole‐cell current of TRPA1 channel was inhibited with an IC_50_ of 54.3 ± 1.3 µm (*n* = 4). The fluorescent images and kinetics of representative cells were randomly chosen from at least 300 individual cells. Scale bar: 50 µm. Values are presented as means ± S.D. from independent experiments performed in triplicate. **p < 0.01, relative to control group.

First, the screening of TRPA1 modulators is shown in **Figure** **4** and Figure S6, Supporting Information. We found a coumarin B‐304 compound that inhibited the TRPA1 channel (Figure 4). The representative fluorescence enhancement from hundreds of individual cells was decreased after administration of the B‐304 on the microfluidic chip (Figure 4a–d). The FlexStation 3 assay result showed that the B‐304 dose‐dependently decreased the calcium fluorescence signals through the TRPA1 channel (Figure 4e). The whole‐cell patch‐clamp recordings further confirmed its inhibitory effect with an IC_50_ value of 54.3 ± 1.3 µm (*n* = 4) and a Hill coefficient of 1.1 (Figure 4f,g). In contrast, false‐positives such as MXL‐6, A‐18, and A‐31 from the FlexStation 3 assay were observed to be negatives in the microfluidic assay (Figure S6, Supporting Information).

We further carried out the screening of TRPV1–V4 modulators (Figures S7–S10, Supporting Information). We identified two coumarin derivatives A‐1 and A‐18 that potently inhibited TRPV2 channel (Figure S8, Supporting Information). The whole‐cell patch‐clamp recordings confirmed their dose‐dependent inhibition of TRPV2 channel with IC_50_ values of 49.06 and 57.51 µm, respectively. The A‐1 also had a weak inhibitory effect on TRPV3 channel with an IC_50_ value over 100 µm (Figure S9, Supporting Information). The coumarin derivative A‐31 also exhibited an inhibitory effect on TRPV3 in the patch‐clamp assay. However, its inhibitory effect was not detected using the microfluidic method, likely due to the fact that the fluorescent calcium assay is an indirect measurement of TRP channel activities, whereas the patch‐clamp recording as a golden standard directly measures ionic current flowing across cell membrane.

The screening results from the three methods are summarized in **Table** **1**. In this small pilot screen (≈1000 samples), 21 out of screened samples were identified from the primary screening in the FlexStation 3 assay. However, only 4 out of the 21 positive results were cherry‐picked from the secondary screening in the single‐cell microfluidic method. The on‐chip screening results were all verified by the patch‐clamp assay except for the inhibitory effect of the A‐31 on TRPV3 channel. Therefore, the single‐cell arrayed microchip dramatically reduces the false‐positive/negative results of the conventional FlexStation 3 screening from 76.2% (16/21) to 4.8% (1/21). More importantly, the four active natural products from *Murraya* plants were reported for the first time for their potential inhibition on the TRP channel activities, including B‐304 on TRPA1, A‐1 on TRPV2, A‐18 on TRPV2, A‐1 on TRPV3, and A‐31 on TRPV3. The result also demonstrated that simultaneously tracking fluorescent kinetics of more than 300 individual cells was sufficient for reliable screening, even though more cells can be better.

**Table 1:** The screening results from three different methods

Channel	Compounds	FlexStation 3	Microfluidics	Patch‐clamp
TRPA1	MXL‐6	**↑**	×	×
	A‐18	**↓**	×	×
	A‐31	**↓**	×	×
	B‐304	**↓**	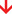	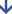
TRPV1	MXL‐38	**↓**	×	×
	A‐1	**↓**	×	×
	ISO	**↓**	×	×
TRPV2	A‐1	**↓**	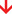	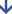
	A‐18	**↓**	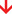	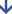
	A‐31	**↓**	×	×
TRPV3	MXL‐6	**↓**	×	×
	A‐1	**↓**	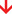	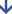
	A‐18	**↓**	×	×
	A‐31	**↓**	×	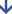
	ISO	**↓**	×	×
TRPV4	ISO	**↓**	×	×
	STR	**↓**	×	×
	DIC	**↓**	×	×
	FRA	**↓**	×	×
	A‐1	**↓**	×	×
	A‐18	**↓**	×	×

**↑** represents agonist; **↓** represents antagonist; × represents inactive. The active results from the single‐cell microfluidic assay are labeled in red, and the active results from the patch‐clamp validation are labeled in blue. The results are from three independent screens.

### 2.6 Antinociceptive Activity of TRPA1 Inhibitor B‐304 In Vivo

**Figure 5:**
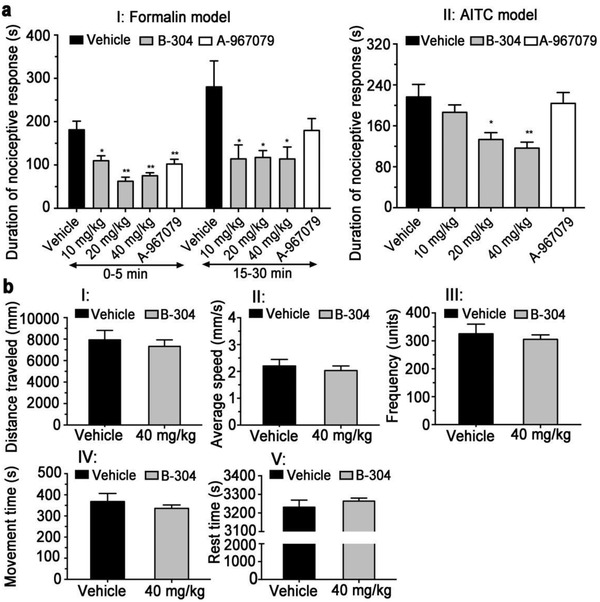
Effects of B‐304 on inhibition of inflammatory pain and locomotor activity in ICR mice. a) Duration of nociceptive response was measured following injection of TRPA1 agonists, formalin (I) or AITC (II). Animals were pretreated with vehicle (black), B‐304 at various doses (grey), or A‐967079 (white). Phase I (0–5 min) and phase II (15–30 min). b) The B‐304 had no effect on locomotor activities including distance travelled (I), average speed (II), activity frequency (III), movement time (IV), and rest time (V); *n* = 8. Values are presented as means ± S.D. *p <0.05, **p < 0.01, relative to vehicle group.

Sustained TRPA1 activation is associated with several pain‐related pathophysiological conditions in vivo.^[^ ^40^ ^]^ To further test the assay reliability, we investigated the effect of the TRPA1 inhibitor B‐304 on pain behaviors in vivo.^[^ ^41^ ^]^ Intragastric administration of the B‐304 before formalin injection into mouse hind paw decreased the total time of flinching and licking observed in both phases I and II (**Figure** **5**a–I), as compared with another TRPA1 agonist A‐967079 that mainly reduced the antinociceptive activity in phase I as reported.^[^ ^42^ ^]^ Similarly, the B‐304 also reversed an AITC‐induced pain in a dose‐dependent manner (Figure 5a‐II).

We further assessed the effect of the B‐304 on locomotion. As shown in Figure 5b, the B304 had no effect on locomotor activity using open field activity measurement. Specifically, the parameters of locomotor activity showed no significant differences between the B‐304 group and the vehicle group, including distance travelled, average speed, activity frequency, movement time, and rest time, respectively. The in vivo results presented here indicated that the TRPA1 inhibitor B‐304 blocked the pain responses without central inhibition.

## 3 Discussion

The goal of this study was to develop a robust microfluidic assay in single‐cell profile for precise screening of TRP channel modulators. This is the first microfluidic assay in single‐cell profile for real‐time monitoring of TRP channel activities, and we have successfully identified four novel TRP channel antagonists of B‐304, A‐1, A‐18, and A‐31.

The single‐cell microfluidic chip developed in this study has its own values for drug screening and ion channel pharmacology study. First, the microfluidic method exhibits a significant reduction of false‐positive/negative rate of the conventional FlexStation 3 screening from 76.2% to 4.8%. Compared with the FlexStation 3 assay, the microfluidic assay improves accuracy and reproducibility of screening results due to full consideration of single‐cell heterogeneity. The fluorescent calcium signals exhibit different patterns in timing and response of individual cells. Cells without fluorescence response are excluded during the on‐chip visualization of the calcium signal. However, the conventional calcium imaging apparatus such as FlexStation 3 can only detect fluorescence signals without obtaining image of population cells in each well of microplate, and the ensemble measurement of FlexStation 3 method also does not reflect the heterogeneity of individual cells. Second, the passive pumping method enables repeated compound administration that is missing in FlexStaion 3 assay without elution process and it facilitates precise and operationally simple delivery of stimuli with low shear stress. Third, the microfluidic chip with closed channel is particularly suitable for screening of trace natural products due to its low sample consumption of 4 µL volume in one test, whereas the FlexStaion 3 assay or patch‐clamp system usually uses compounds with 100 µL or 10 mL volume, respectively. Taken together, this microfluidic platform is suitable for target‐based compound screening in single‐cell format.

However, it is noted that this merit does not supersede the gold standard of patch‐clamp due to its indirect measurement of ion channel activities. This microfluidic device was manufactured from commonly used material of PDMS, and the issue with absorption of small and hydrophobic molecules for PDMS should have little concern in the laboratory, although thermoplastic material can be used for chip industrialization and reuse instead of PDMS.

Using the developed microfluidic assay in single‐cell profile, we screened and identified four novel coumarin type ingredients, B‐304, A‐1, A‐18, and A‐31, isolated from the *Murraya* species which could potently inhibit the TRP channels. *Murraya* plants have been widely used as medical herbs for relief of psychogenic and somatoform pain.^[^ ^43^ ^]^ Our findings not only demonstrate the mechanism of action for the novel compounds, but also point to the possibility of identifying structurally diversified natural products targeting TRP channels. One of the positive hits, B‐304, inhibits TRPA1 channel and attenuates the formalin‐ and AITC‐evoked pain sensitization behavior in vivo. TRPA1 channel as a pain target is involved in formalin's pain‐producing action in two phases.^[^ ^41^ ^]^ Phase I is thought to result from direct activation of primary afferent sensory neurons for neurogenic inflammation, whereas phase II is dependent on peripheral inflammation and central sensitization of pain.^[^ ^44^ ^]^ We found that the B‐304 decreased pain‐related flinching and licking in both phases without central inhibition. To date, A‐967079, HC‐030031, and AP18 are the most commonly used TRPA1 tool antagonist for studying TRPA1 biology in vivo and in vitro.^[^ ^45^ ^]^ However, A‐967079 is very expensive and mainly reduced the antinociceptive activity in phase I.^[^ ^42^ ^]^ HC‐030031 inhibited several proteins involved in pain signaling and exhibited undesirable pharmacokinetic properties.^[^ ^46^, ^47^ ^]^ Selective antagonist of AP18 was not systematically bioavailable through oral or intraperitoneal dosing.^[^ ^48^ ^]^ Here, we report the discovery of the B‐304, a cost‐effective and intragastrically bioavailable TRPA1 antagonist. The B‐304 may serve as a tool compound or lead for studying TRPA1 physiology and pharmacology, although its selectivity and pharmacokinetic properties require further investigation.

In summary, we developed a microfluidic assay in single‐cell profile for screening of ion channel modulators using a minimum amount of compounds. Using this platform, we identified a TRPA1 inhibitor B‐304 that exhibits a developmental potential for in vivo pain relief. This assay is an efficient supplement between the conventional assays of mini‐FLIPR‐based FlexStation 3 and patch‐clamp electrophysiology. The system can be further scaled up to adapt automatic high‐throughput screening in a 96‐well or 384‐well microplate format. Automated reagent dispensers can carry out cell seeding and drug loading, while cell imaging can be achieved by addressable microscope. We envision our platform to be applied to broad single‐cell analysis, such as cellular signaling dynamics, pharmacodynamics, stem cells, and cancer research.

## 4 Experimental Section

##### Design and Fabrication of the Microfluidic Chip

The PDMS microchip was designed in AutoCAD software and fabricated using standard soft lithography fabrication technology.^[^ ^23^ ^]^ SU‐8 2050 negative photoresist (MicroChem) was used to fabricate 40 µm thick micropillars and 100 µm thick micropattern on silicon wafers. A closed microchannel on the top layer had 1 mm width, 100 µm depth, and 10 mm length. The wafers were then treated with tridecafluoro‐(1,1,2,2‐tetrahydrooctyl)‐1‐trichlorosilane saline (Sigma‐Aldrich). Next, PDMS (10A: 1B; Dow Coring) was poured onto the photoresist masters and heated at 70 °C for 1 h to achieve a fully cross‐linked PDMS replica‐molded. The two pieces of PDMS were peeled off, holes punched, and plasma bonded irreversibly (Harrick Plasma, PDC‐32G) to form the microfluidic chip. It is believed that the microchip would be suitable for long‐term cell culture with high viability due to biocompatible PDMS material.^[^ ^49^, ^50^ ^]^

##### Cell Culture, Transient Transfection, and Staining

HEK‐293T cell line was purchased from Peking Union Medical College, Cell Bank (Beijing, China). The HEK‐293T cells were cultured in Dulbecco's modified eagle medium supplemented with 10% fetal bovine serum, penicillin (100 U mL^−1^), and streptomycin (100 µg mL^−1^) at 37 °C under a humidified atmosphere of 5% CO_2_ and 95% air. The cells were transiently transfected using lipofectamine 2000 (Invitrogen) with 4 µg human *TRPA1* and *TRPV1*–*4* cDNAs, respectively. Experiments were performed between 18 and 36 h after transfection. Then, the cells were stained with indicators from the Cal‐520 PBX calcium assay kit for 75 min at 37 °C h in the presence of 2.5 mm probenecid.

##### Operation of Microfluidic Device and Characterization of Media Replacement

The microfluidic chip was primed with HBSS with 2% bovine albumin (Sigma‐Aldrich) to prevent non‐specific cell adhesion to the device surface and to remove bubbles. A 4 µL cell suspension at a density of 1 × 10^7^ cells mL^−1^ was loaded into the single‐cell trap module by pipetting and settled by gravity for 2 min. To increase the single‐cell trapping efficiency, multiple loading procedures were performed with a cell suspension at a density of 5 × 10^6^ cells mL^−1^ as desired. The excess cells were removed and the device was rinsed with PBS solution. Parameters were optimized in order to obtain the highest single‐cell trapping efficiency and desired throughput.

The medium delivery was achieved by passive pumping using hand pipettes.^[^ ^21^ ^]^ Figure S3a, Supporting Information, shows procedure of switching media from solution A (green) to solution B (purple) on the microfluidic chip. A 30 µL droplet of HBSS was dispensed at outlet ports. After 10 s, a 4 µL droplet containing the solution A were dispensed at the inputs. After 60–70 s, a 4 µL droplet containing the solution B were dispensed at the inputs to completely replace the solution A.

To quantify time‐lapsed media replacement in the microchannel, 4 µL droplets containing deionized (DI) water or fluorescein isothiocyanate (FITC, 1.0 µm concentration) were loaded into the microchannel alternately by passive pumping. Sequences of fluorescence images of replacing FITC with DI water were obtained at a speed of 5 Hz with an exposure time of 100 ms. Flow rate and transition time were adjusted according to the volume of the droplet in the inlet from three independent experiments. Flow profile within the microfluidic chip during the media replacement was simulated using a finite element analysis software (COMSOL MultiPhysics 3.5a). The simulations presented here used Navier–Stokes equation for incompressible flow and convection‐diffusion equation. As boundary conditions, a laminar inflow was set at the inlets, zero pressure at the outlets, and no slip on the walls. The cell viability assessment was performed using a Live/Dead assay kit (Invitrogen) before and after the media replacement.

##### Monitoring TRP Channel Activity on the Microfluidic Chip

The solutions A and B for feasibility assessment of the microchip are listed in Table S2, Supporting Information. To test repetitive compound administration on the TRPV3‐transfected HEK‐293T cells, 200 µm 2‐APB and HBSS were administered repeatedly for three times, as well as a series of concentrations of 2‐APB (12.5, 50, 200 µm) in sequence. The device was mounted on microscope stage and the calcium dynamics of single cells were recorded at 1 Hz.

##### Screening of TRP Channel Modulators

Approximately 200 small molecules were screened against cells expressing five TRP channel subtypes (TRPA1 and TRPV1–4). A primary screening was preliminarily run using the FlexStation 3 assay. The transfected HEK‐293T cells were seeded at a density of ≈30 000 cells per well in 96‐well black‐walled plates covered with poly‐d‐lysine. Solutions A and B were added into well at 17 s and 100 s, respectively (Table S3, Supporting Information). Detection time of the whole process was 180 s. Fluorescence intensity from a population of cells was measured at an interval of 1.6 s with an excitation wavelength at 485 nm and an emission wavelength at 525 nm.

The active results from the primary screening were further conducted for a secondary screening using the single‐cell arrayed microchip and verified using the gold standard of patch‐clamp. The total screening time on the microchip was ≈8 min, including about 6 min of cell seeding and 2 min for drug loading and imaging. Whole‐cell recordings were performed using a HEKA EPC10 amplifier with Patchmaster software (HEKA). Patch pipettes were pulled with borosilicate glass using a puller (DMZ‐Universal) and fire‐polished to a resistance of 3–5 megohms. Membrane potential was held at 0 mV and current in response to 400 ms voltage ramps from −100 to +100 mV.

##### Animals

All experiments involving animals were performed in accordance with the Institutional Animal Care and Use Committee protocols. Adult male albino Swiss mice (ICR, average weight ≈18–22 g) were used for the experiments.

For formalin test, the mice were allowed to habituate to Plexiglas chambers for at least 15 min. One hour later after drug administration, the formalin (20 µL of 1% formalin, diluted in saline) was injected into the pelma of the left hind paw of the mouse. The animal was put into a Plexiglas chamber where movement of the formalin‐injected paw was recorded using a digital video recorder (Hikvision Digital Technology). The time of paw flinching and licking was counted by seconds over the next 30 min. Phases were defined as follows: Phase I (0–5 min) and phase II (15–30 min). AITC‐induced nociceptive responses were carried out identically and animals were injected with the AITC (20 µL of 0.2% w/w AITC, diluted in 92% gingili oil) instead of formalin. The time of paw flinching and licking was counted by seconds over the next 20 min. The animals were divided into five groups, including the B‐304 groups (10, 20, 40 mg kg^−1^ diluted in 0.5% w/v sodium carboxymethylcellulose), A‐967079 group (positive control, 16 µg/20 µL diluted in saline), and the control group. The control group received equivalent injections of the respective vehicle solutions.

Locomotor function was tested by using an open field activity monitoring system (JLBehv). Effect of compound B‐304 with 40 mg kg^−1^ was assessed 60 min after a single intragastric administration (*n* = 8), calculating the distance travelled (mm), average speed (mm s^−1^), activity frequency (unit), movement time (sec), and rest time (sec) per mouse and group. After the assay, the animals were euthanized by cervical dislocation.

##### Imaging and Data Analysis

The fluorescent calcium images of single cells were recorded under a fluorescence microscope (OLYMPUS IX73) with a 10× objective (UPlanFLN10 × 0.30 Ph1 ∞/‐/FN26.5) and a camera (DP80, 2CCD cooled digital camera) at an exposure time of 500 ms. The fluorescent calcium profiles of single cells were quantified by using Image Pro Plus 6.0 software (Media Cybernetics). GraphPad Prism 6 (GraphPad) was used for statistical analysis. At least 300 individual cells were measured for each sample. The intracellular calcium fluorescence intensity of single cells at each time point was recorded as relative fluorescence units (RFU). The calcium fluorescence enhancement (FE) was calculated by the following formula: FE = the highest RFU value after adding solution A/B − the basal RFU value before adding solution A/B. The agonist's criterion was fluorescence enhancement of compounds above that of the DMSO group after addition of solution A. The antagonist's criterion was the fluorescence enhancement of compounds lower than that of the positive control (AITC/CAP/2‐APB/GSK) after the addition of solution B. Inhibition rate was calculated by the following formula:
(1)
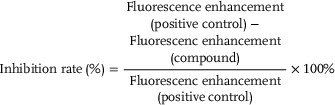



The bigger the difference of the fluorescence enhancement between the compounds and the DMSO group, the stronger the agonist or antagonist. All data were presented as mean ± S.D. of at least three independent experiments. One‐way analysis of variance (ANOVA) was used to determine the statistical significance of the different treatment groups. A value of *p* < 0.05 was used to indicate statistically significant difference. EC_50_ and IC_50_ values are the concentrations for half‐maximal effects.

## Conflict of Interest

The authors declare no conflict of interest.

## Supporting information

Supporting Information
