# Supplementary material for: A Precise Microfluidic Assay in Single‐Cell Profile for Screening of Transient Receptor Potential Channel Modulators
Source: Adv Sci (Weinh). 2020 Apr 19;7(11):2000111. doi: 10.1002/advs.202000111 (PMC7284206; doi:10.1002/advs.202000111)
Supplement: Supplementary file 1 — Supporting Information [file ADVS-7-2000111-s001.pdf]

Copyright WILEY-VCH Verlag GmbH & Co. KGaA, 69469 Weinheim, Germany, 2020.

Supporting Information

A precise microfluidic assay in single-cell profile for screening of TRP channel modulators

Xiaoni Ai, Yang Wu, Wenbo Lu, Xinran Zhang, Lin Zhao, Pengfei Tu, KeWei Wang* and Yong Jiang**

Table of Contents

1. **Figure S1.** Parameter optimization for single-cell trapping in the microwells……..S-3
2. **Figure S2.** Media replacement by passive pumping…………………………….….S-4
3. **Figure S3.** Whole-cell currents of non-transfected and TRP channel expressed cells………………………………………...……………………………….….S-6
4. **Figure S4.** Methodological validation of the microchip for monitoring intracellular calcium response of individual cells to TRPV3 modulators…..……………………S-7
5. **Figure S5.** Structures of the active compounds obtained from the primary screening of the FlexStation 3 assay..…………………….………….………..…....……S-8
6. **Figure S6.** The activity screening for TRPA1 modulators..……………..……...…S-9
7. **Figure S7.** The activity screening for TRPV1 modulators.….................................S-10
8. **Figure S8.** The activity screening for TRPV2 modulators..……………….....…..S-12
9. **Figure S9.** The activity screening for TRPV3 modulators on the

microchip…………………………………………………………………………..S-14

10. **Figure S10.** The activity screening for TRPV4 modulators on the microchip... ……………………………………………………………….…S-15

11. **Table S1.** The representative screening results from the primary screening by the conventional Ca^{2+} imaging method of FlexStation 3 assay..................................…S-16

12. **Table S2.** The Solution A and B in the DMSO group (DG) and the compound group (CG) for feasibility assessment of the microchip..…S-16

13. **Table S3.** The Solution A and B in the DMSO group (DG) and the compound group (CG) for screening of TRP channel modulators………………………………......S-17

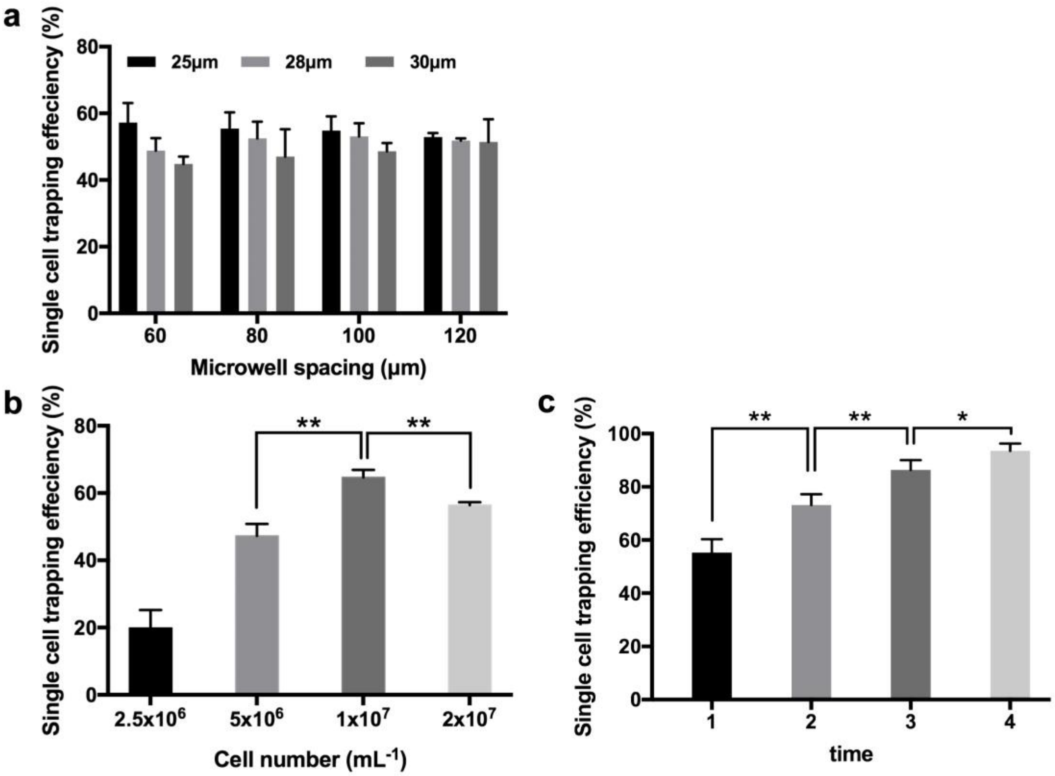

Figure S1. Parameter optimization for single-cell trapping in the microwells. **a)** Effects of various microwell spacings and diameters on single-cell trapping efficiency. Under the same microwell spacing, the single-cell trapping efficiency slightly decreased with the increased microwell diameter. There was no discernible difference in the single cell-trapping efficiency among the microwell arrays with different microwell spacings. **b)** Effects of cell seeding density on single cell trapping efficiency. Increasing the cell seeding density resulted in the increased single cell trapping efficiency. **c)** Effects of loading times on single cell trapping efficiency. Increasing the cell loading times resulted in the increased single cell trapping efficiency at the risk of multiple cells occupancy in a single microwell. Values are presented as means ± S.D. from independent experiments performed in triplicate. *p <0.05, **p < 0.01, relative to control group.

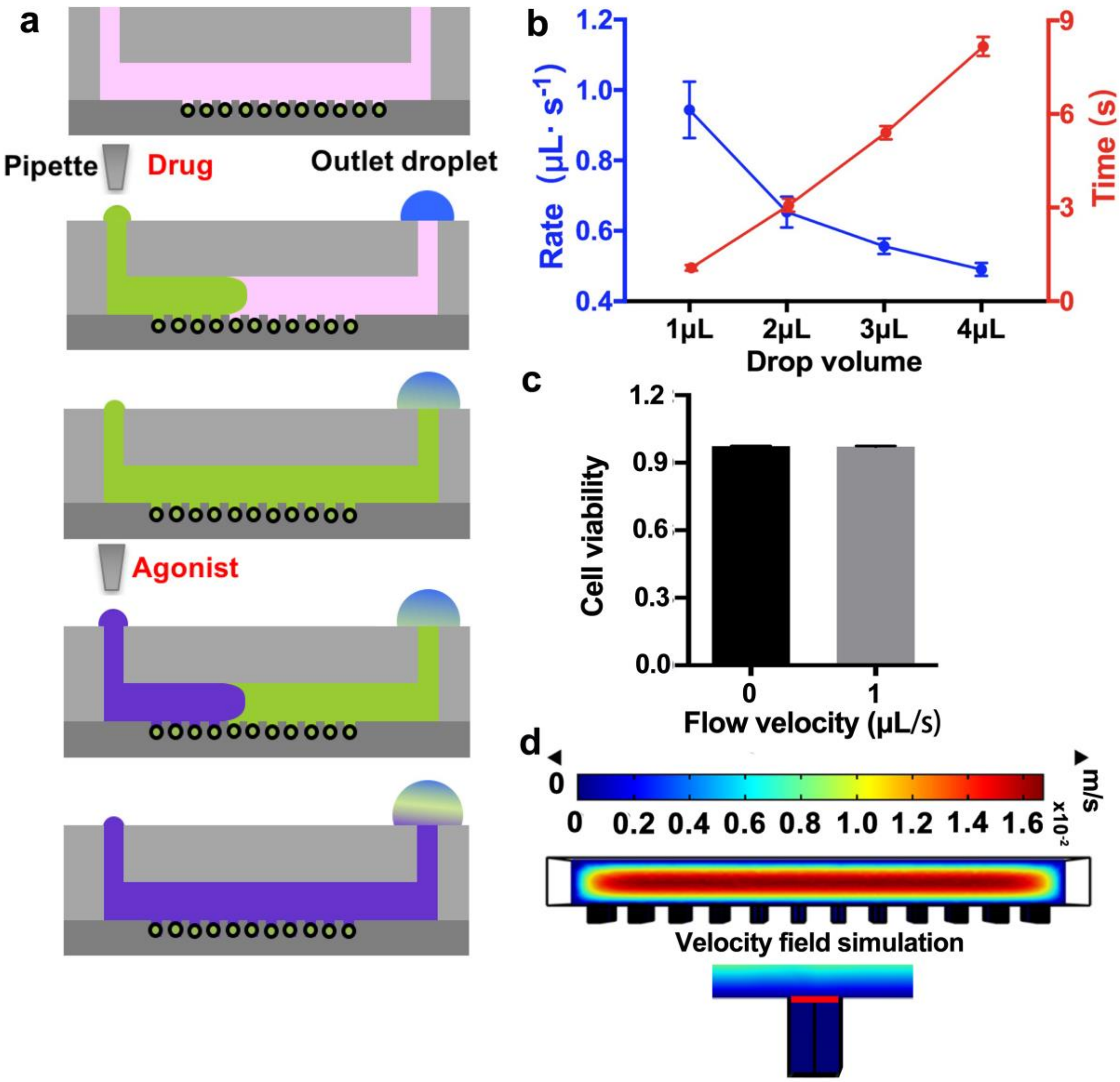

Figure S2. Media replacement by passive pumping. **a)** Schematic diagram of procedure of switching media from Solution A (drug as an example, green) to Solution B (agonist as an example, purple) by passive pumping. **b)** Flow rate and transition time of complete removal fluorescein by DI water *versus* drop volume. **c)** Cell viability test was conducted before and after fluid exposure at 1 μL s^{-1} for 180 s. Live cells were stained in green and dead cells stained in red using a Live/Died assay kit. Viability was calculated using green cell number divided by total cell number. There were no statistically significant

differences in the cell viability before and after fluid exposure. **d)** Computational analysis of fluid velocity contours along the microchannel with pumping the fluid at 1 $\mu L \cdot s^{-1}$. The maximum flow velocity on the top of the microwell as labeled in red was 6.5×10^{-4} m/s, corresponding to shear stress of 3.5×10^{-2} Pa. Results are presented as means ± S.D. from independent experiments performed in triplicate.

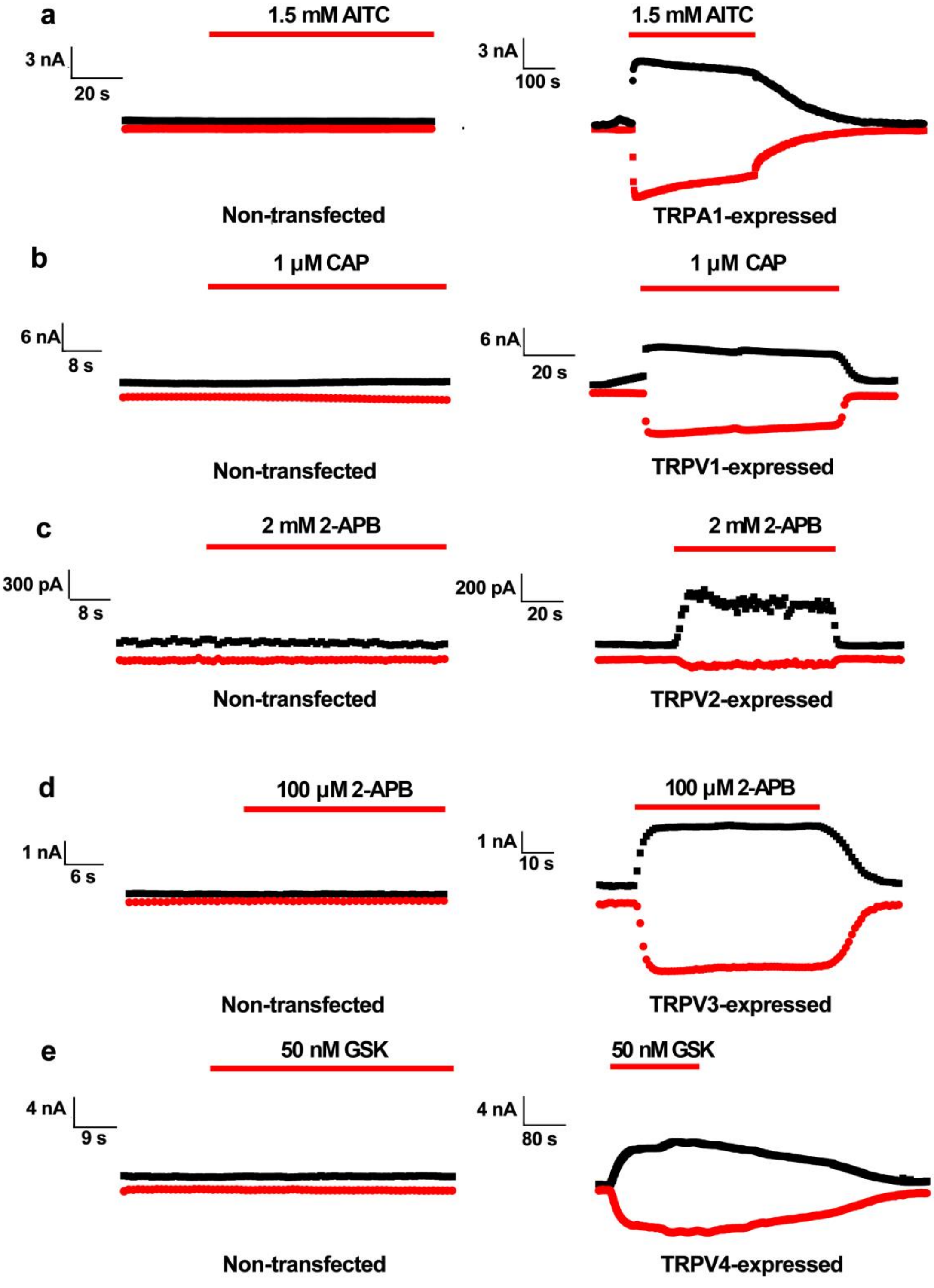

Figure S3. Whole-cell currents of non-transfected and TRP channel expressed cells.

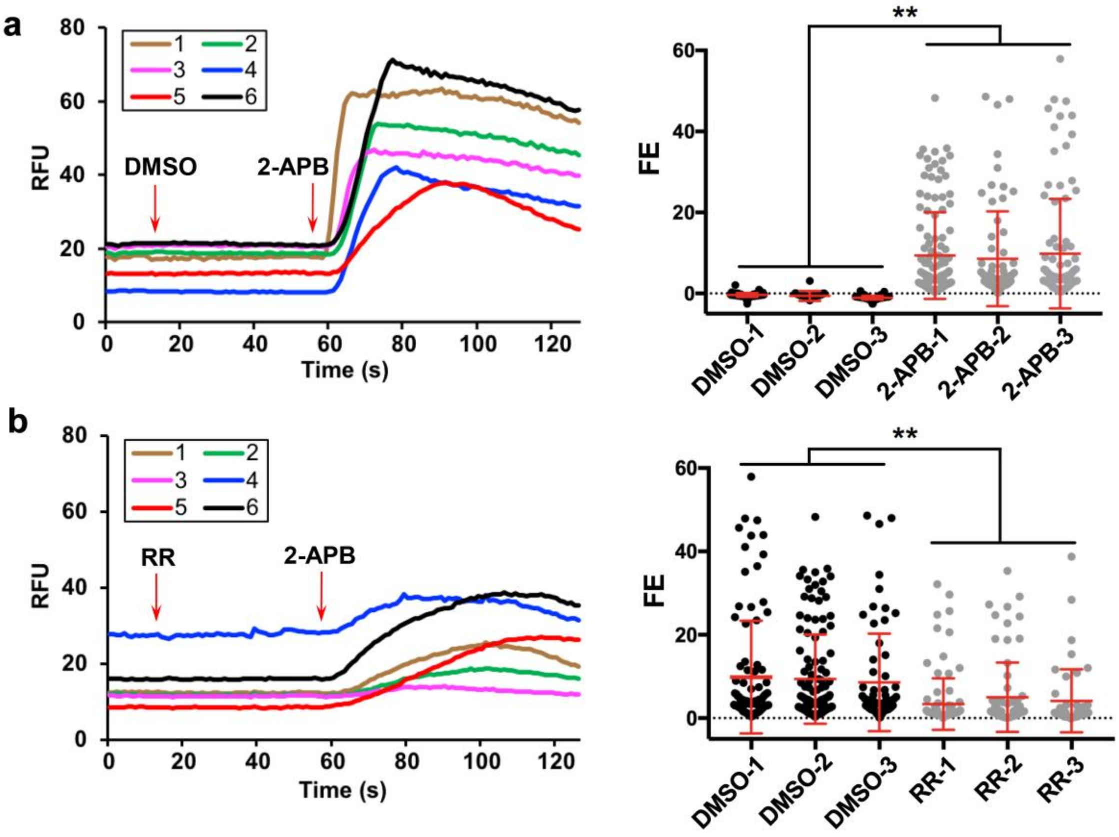

Figure S4. Methodological validation of the microchip for monitoring intracellular calcium response of individual cells to TRPV3 modulators. **a, b)** Representative kinetics of calcium fluorescence and fluorescence enhancement distribution from hundreds of the individual cells in **a)** the 2-APB group and **b)** the RR group. The representative individual cells were randomly chosen from at least 300 individual cells. Values are presented as means ± S.D. from independent experiments performed in triplicate. $^{**}p < 0.01$, relative to control group.

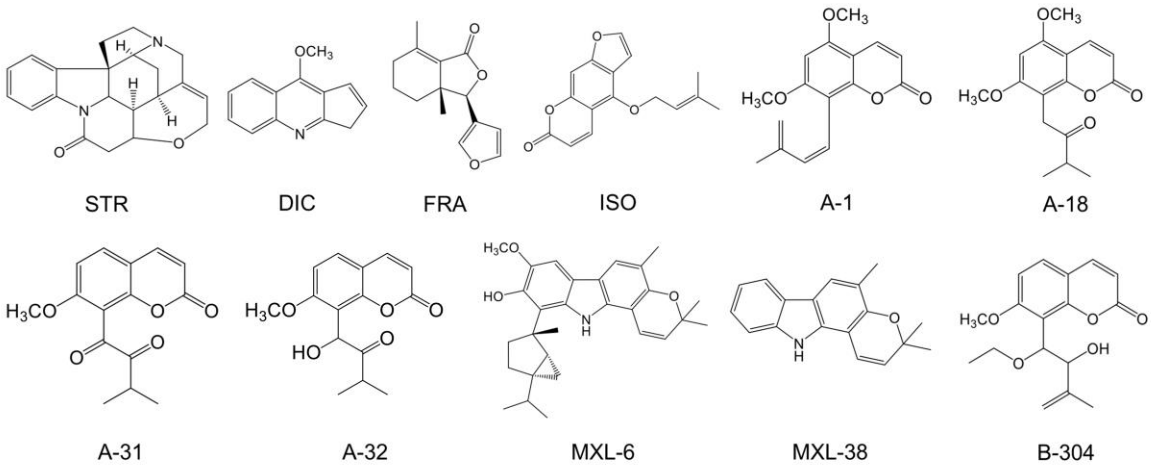

Figure S5. Structures of the active compounds obtained from the primary screening of FlexStation 3 assay, including strychnine (STR), dictamnine (DIC), fraxinellone (FRA), isoimperatorin (ISO), five coumarins of A-1, A-18, A-31, A-32 and B-304, as well as two carbazole alkaloids of MXL-6 and MXL-38.

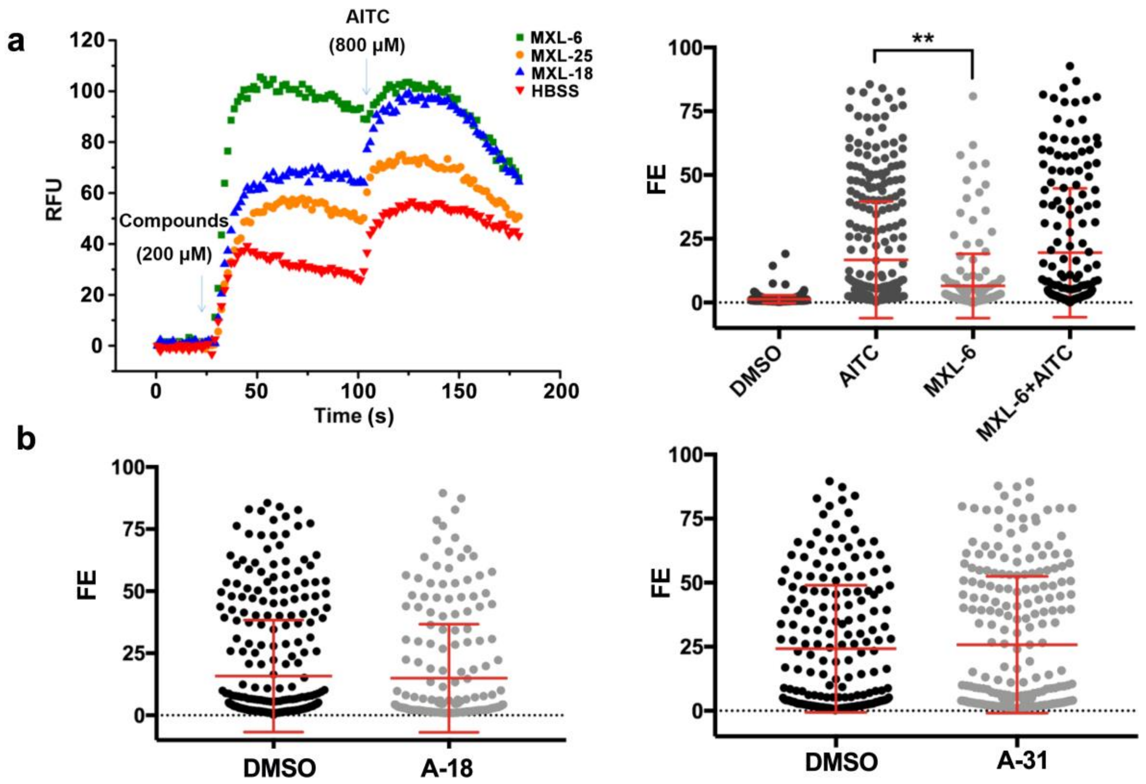

Figure S6. The activity screening for TRPA1 modulators. **a)** The TRPA1 agonist screening. The agonistic effect of MXL-6 exhibited superiority with the strongest fluorescence enhancement using the FlexStation 3 assay. However, the agonistic effect of the MXL-6 was not observed on the microfluidic chip. **b)** The TRPA1 antagonist screening on the microfluidic chip. The inhibitory effects of A-18 and A-31 were not observed without any significant differences of the fluorescence enhancement between the compound group and the DMSO group. The fluorescence enhancements from hundreds of individual cells were measured. Values are presented as means ± S.D. from independent experiments performed in triplicate. $^{**}p < 0.01$, relative to control group.

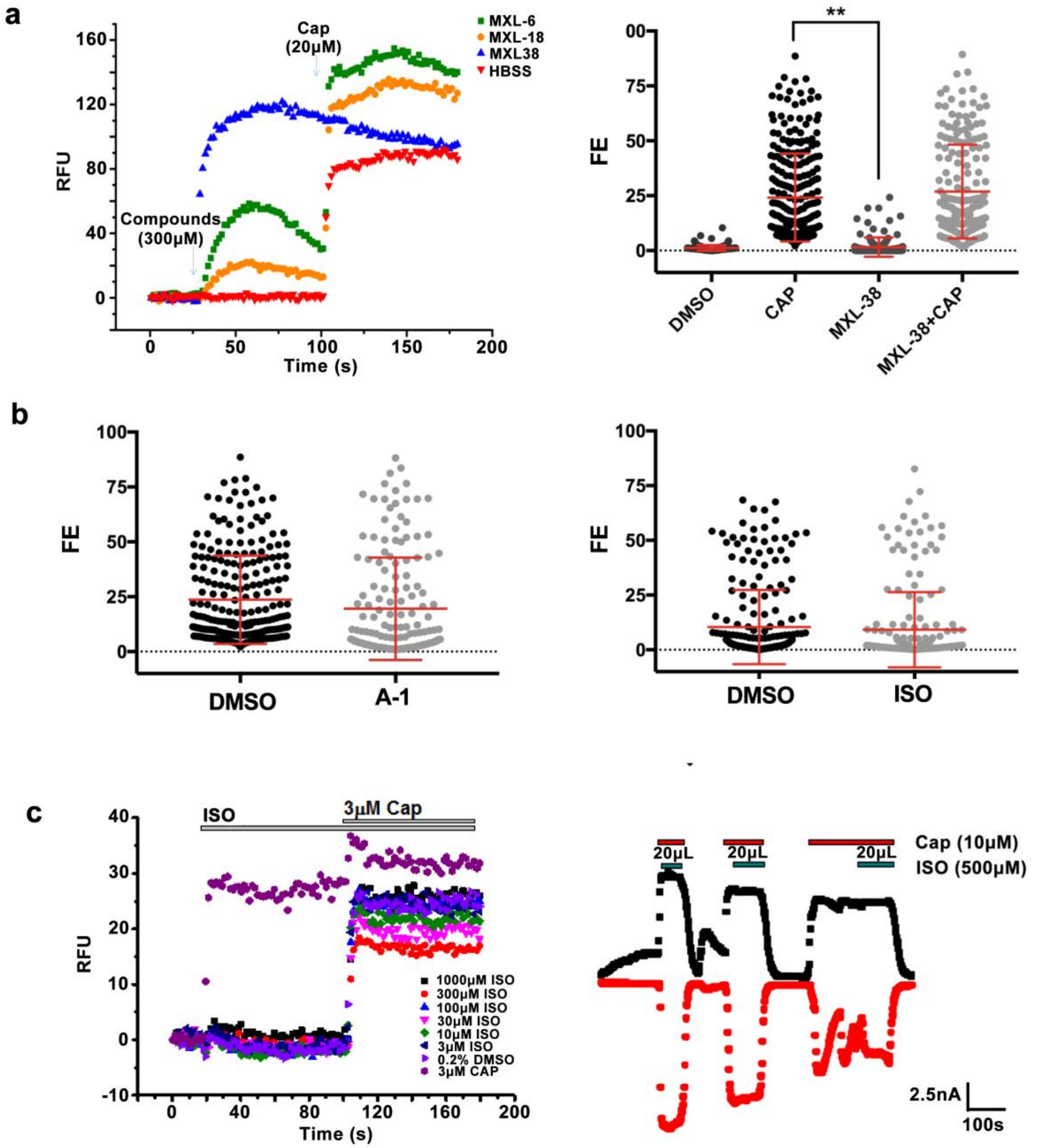

Figure S7. The activity screening for TRPV1 modulators. **a)** The TRPV1 agonist screening. The MXL-38 showed the strongest agonistic effect with the strongest fluorescence enhancement using the FlexStation 3 assay. However, the agonistic effect of the MXL-38 was not observed on the microfluidic chip. **b)** The TRPV1 antagonist screening on the microfluidic chip. The inhibitory effects of A-1 and ISO were not

observed without any significant differences of the fluorescence enhancement between the compound group and the DMSO group. **c)** The antagonist activity of ISO tested using the FlexStation 3 assay and patch clamp. The ISO showed dose-dependently decreased the calcium fluorescence signals on TRPA1 channel from the FlexStation 3 assay, however, exhibited no inhibitory activity using patch clamp (500 μM ISO). The fluorescence enhancements from hundreds of individual cells were measured. Values are presented as means ± S.D. from independent experiments performed in triplicate. ** $p < 0.01$, relative to control group.

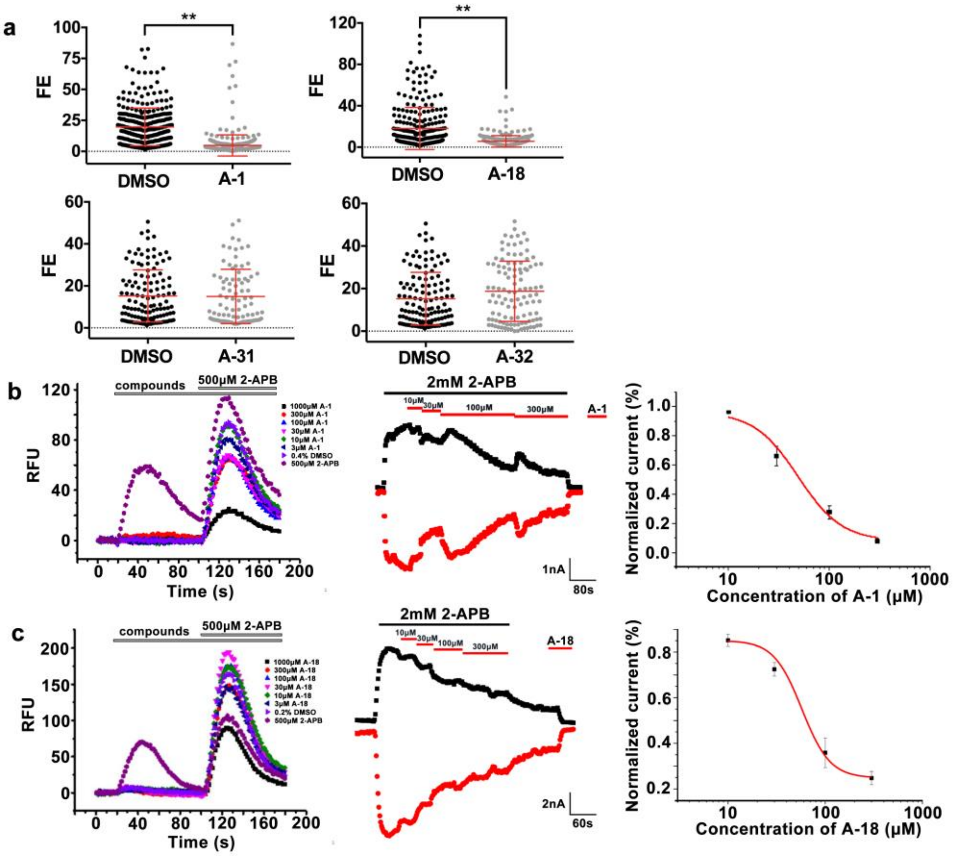

Figure S8. The activity screening for TRPV2 modulators. **a)** The TRPV2 antagonist screening on the microfluidic chip. The A-1 and A-18 showed inhibitory effects with the decreased fluorescence enhancements after the compound administration. However, the A-31 and A-32 exhibited no agonistic effects. **b–c)** The antagonist activities of **b)** A-1 and **c)** A-18 tested using the FlexStation 3 assay and patch clamp. The A-1 and A-18 showed the dose-dependent inhibition on TRPV2 channel from the FlexStation 3 assay. The whole-cell currents of TRPV2 channel were inhibited by increasing the concentrations of A-1 and A-18 from 10 μM to 300 μM. The fluorescence enhancements from hundreds of individual cells were measured. Values are presented as means ± S.D. from independent experiments performed in triplicate. ***p* < 0.01, relative to control group.

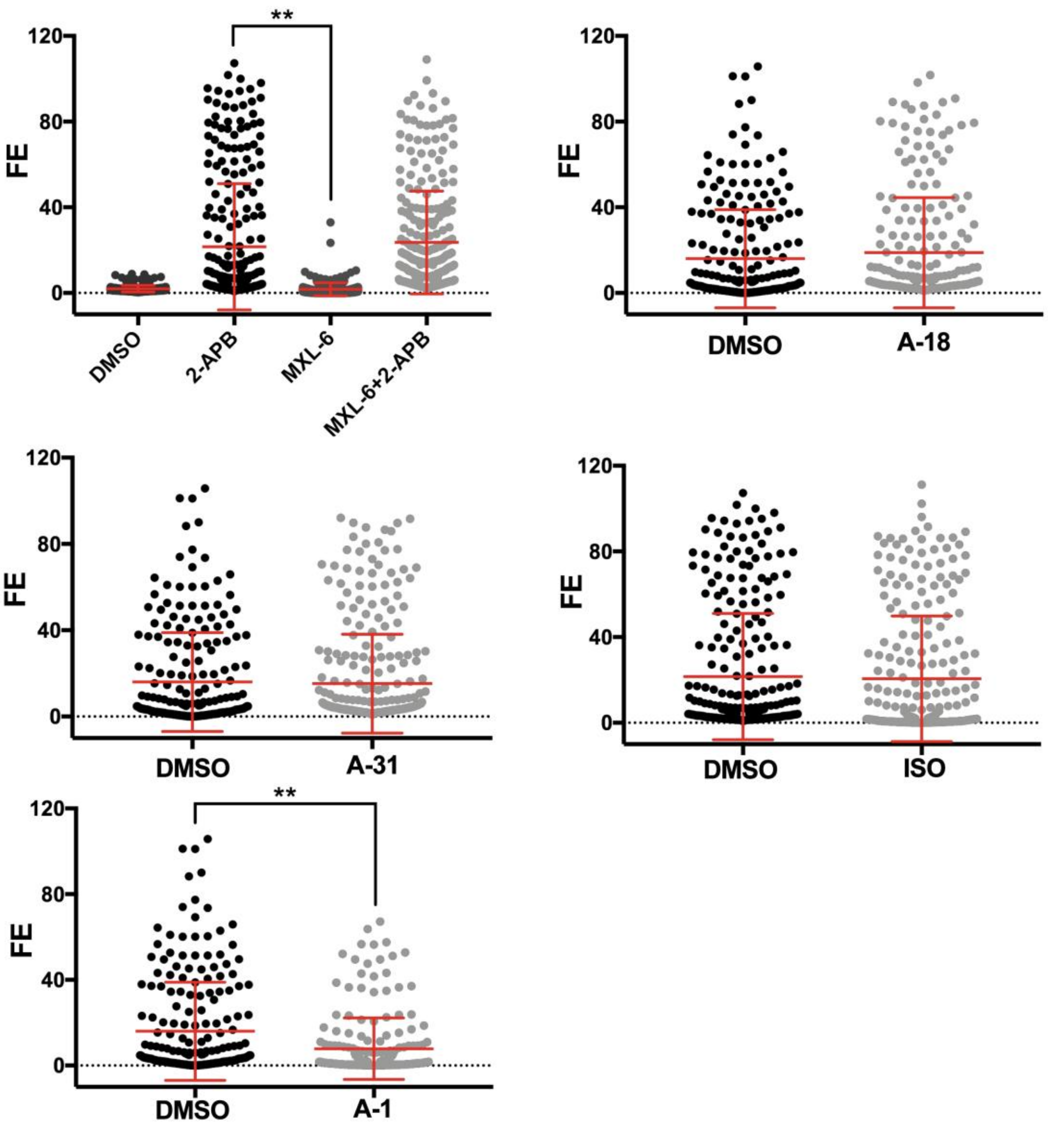

Figure S9. The activity screening for TRPV3 modulators on the microchip. The MXL-6, A-18, A-31 and ISO showed no activity on TRPV3 channel without any significant differences of the fluorescence enhancements between the compound group and the DMSO group. However, the A-1 showed a weak inhibitory effect on TRPV3 channel with a wide variety of the fluorescence enhancement distribution of the individual cells. The fluorescence enhancements from hundreds of individual cells were measured. Values are presented as means ± S.D. from independent experiments performed in triplicate. ***p* < 0.01, relative to control group.

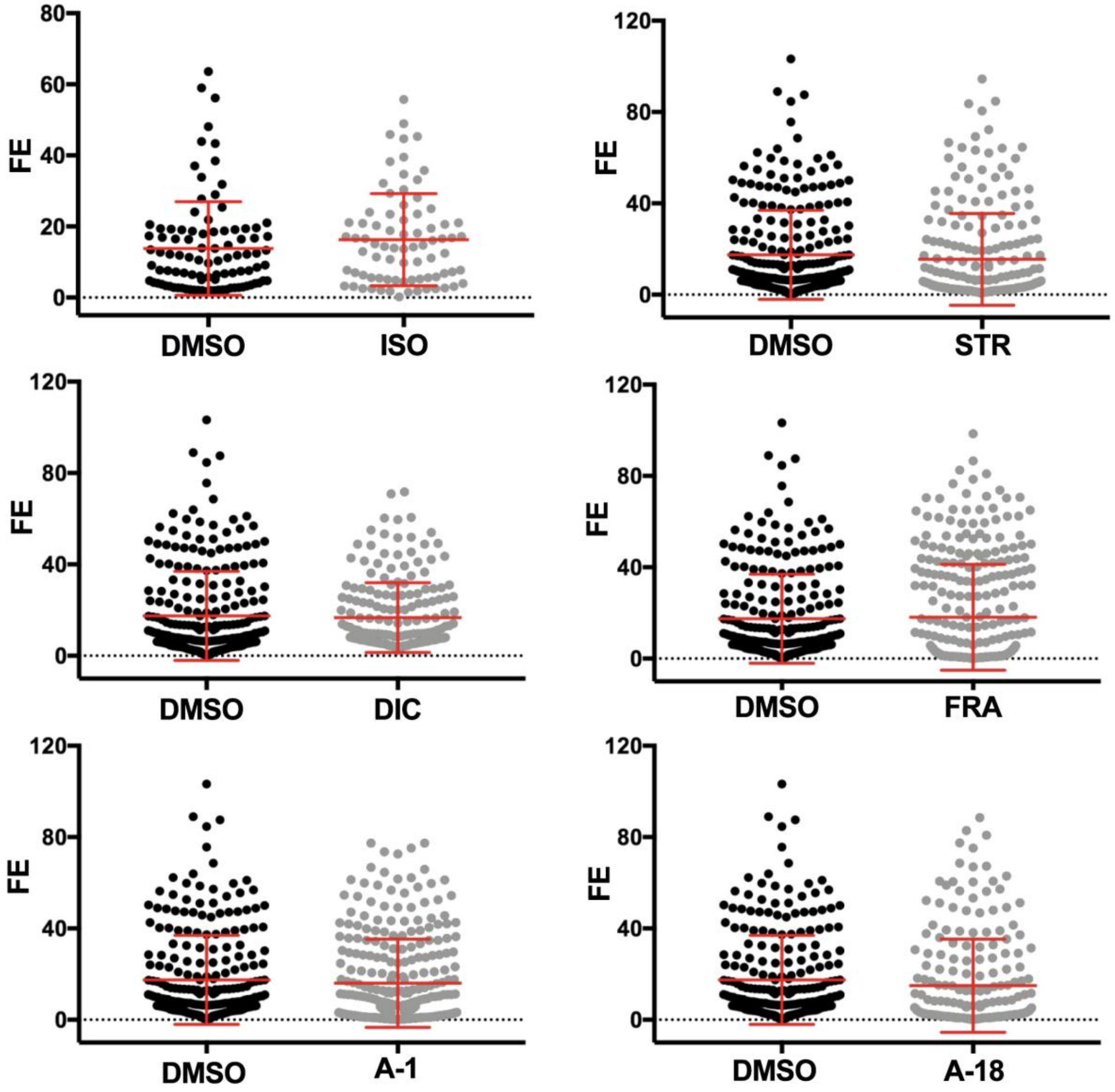

Figure S10. The inhibitory activity screening for TRPV4 modulators on the microchip. The ISO, STR, DIC, FRA, A-1 and A-18 showed no antagonistic effects on TRPV4 channel without any significant differences of the fluorescence enhancement between the compound group and the DMSO group. The fluorescence enhancements from hundreds of individual cells were measured. Values are presented as means ± S.D. from independent experiments performed in triplicate.

Table S1. The representative results from the primary screening by the conventional Ca^{2+} imaging method of FlexStation 3 assay.

化合物	TRPA1	TRPV1	TRPV2	TRPV3	TRPV4
STR	✕	✕	✕	✕	↓ 23% 66%
DIC	✕	✕	✕	✕	↓ 49% 65%
FRA	✕	✕	✕	✕	↓ 58% 49%
ISO	✕	↓ 72% 23%	✕	↓ 17% 73%	↓ 73% 66%
A-1	↓ 27% 45%	↓ 34% 64%	↓ 59% 78%	↓ 66% 83%	↓ 86% 85%
A-18	↓ 59% 56%	↓ 34% 17%	↓ 9% 81%	↓ 51% 8%	↓ 55% 30%
A-31	↓ 70% 31%	✕	↓ 31% 67%	↓ 91% 100%	↓ 48% 43%
A-32	✕	✕	✕	↓ 76% 61%	✕
MXL-6	↑	↑	✕	↑	✕
MXL-38	✕	↑	✕	✕	✕
B-304	↓ 17% 61%	✕	✕	✕	✕

Notes: ↑ represents agonist, ↓ represents antagonist, ✕ represents inactivity. Results were from at least two independent experiments.

Table S2. The Solution A and B in the DMSO group (DG) and the compound group (CG) for feasibility assessment of the microchip.

Modulators	TRP Channel	Solution A (DG)	Solution B (DG)	Solution A (CG)	Solution B (CG)
Activation 2-APB	TRPV1	0.2%DMSO		400 μM 2-APB	
	TRPV3	0.2%DMSO		200 μM 2-APB	
Inhibition RR	TRPV1	0.2%DMSO	400 μM 2-APB	20 μM RR	20 μM RR + 400 μM 2-APB
	TRPV3	0.2%DMSO	200 μM 2-APB	20 μM RR	20 μM RR + 200 μM 2-APB

Table S3. The Solution A and B in the DMSO group (DG) and the compound group (CG) for screening of TRP channel modulators.

**TRP channel**	**Solution A (DG)**	**Solution B (DG)**	**Solution A (CG)**	**Solution B (CG)**
TRPA1	0.2%DMSO	300 μM AITC	X	X + 300 μM AITC
TRPV1	0.2%DMSO	5 μM CAP	X	X + 5 μM CAP
TRPV2	0.2%DMSO	500 μM 2-APB	X	X + 500 μM 2-APB
TRPV3	0.2%DMSO	200 μM 2-APB	X	X + 200 μM 2-APB
TRPV4	0.2%DMSO	0.2 μM GSK	X	X + 0.2 μM GSK
